# Engaging Key Players in Partner Workgroups: A Collaborative Approach to Explanatory Trials in NHs

**DOI:** 10.1093/geroni/igaf122.454

**Published:** 2025-12-31

**Authors:** Jasmine Altizer, Alice Bonner, Shih-Yin Lin, Sonya Jha, Gail Towsley, Jennifer Carnahan, Sheryl Zimmerman

**Affiliations:** New York University, New York, New York, United States; Institute for Healthcare Improvement, Boston, Massachusetts, United States; New York University Rory Meyers College of Nursing, New York, New York, United States; New York University, New York, New York, United States; University of Utah, Salt Lake City, Utah, United States; Indiana University Center for Aging Research, Indianapolis, Indiana, United States; University of North Carolina at Chapel Hill, Chapel Hill, North Carolina, United States

## Abstract

To support the NEXT STEPs Network’s goal of creating a national infrastructure that will address systemic barriers to conducting research in nursing homes, we have established four partner workgroups (i.e., nursing home residents, care partners, nursing home clinical staff and nursing home administrators and leaders). These partner workgroups play a key role in representing essential perspectives for conducting research in nursing homes. Each workgroup consists of approximately 5-8 members and meets three times a year via Zoom. To recruit workgroup members, potential candidates were first identified through NEXT project investigator connections, national organizations, the LTCDC, and other organizations. The New York University team (part of NEXT’s Recruitment and Retention Core) then sent formal invitations outlining the expectations of workgroup members, along with a demographic form to ensure diversity in gender, race and ethnicity, and geographic representation within the workgroups. Workgroup members review and contribute to best practice guidance documents and training materials, advise on pilot grants, and provide feedback on specific interventions for nursing home clinical trials. To obtain input and feedback on specific products/activities, NEXT STEPs Cores (Projects and Training; Recruitment and Retention; Methods, Measures, and Data) complete a standardized online request form, specifying how they would like to receive feedback from the workgroups (e.g., attending a virtual workgroup meeting, receiving an email summary of workgroup recommendations, cognitive interviewing style via Qualtrics, etc.). In this session, we will explore facilitators, barriers, and lessons learned during recruitment and continued engagement of the partner workgroups.

